# Pregnant women’s preferences for and concerns about preterm birth prevention: a cross-sectional survey

**DOI:** 10.1186/s12884-017-1221-z

**Published:** 2017-01-31

**Authors:** Vanessa Ha, Sarah D. McDonald

**Affiliations:** 1 Department of Health Research Methods, Evidence, and Impact, McMaster University, Hamilton, ON Canada; 2 Department of Obstetrics & Gynecology, McMaster University, Hamilton, ON Canada; 3 Department of Radiology, McMaster University, Hamilton, ON Canada

**Keywords:** Preterm birth prevention, Patient preference, Survey, Progesterone, Cerclage, Pessary

## Abstract

**Background:**

Although there is a call for patient-centred prenatal care, women’s preferences for and concerns about preterm birth (PTB) prevention have not been well-studied. Therefore, we conducted a cross-sectional survey to determine women’s preferences for PTB prevention and their likelihood of following their healthcare provider’s recommendations for PTB prevention, as well as factors associated with these responses.

**Methods:**

A piloted self-administered questionnaire was completed by pregnant women who could read English. Data were collected about their preferences for and concerns about PTB prevention, and the likelihood of following their healthcare provider’s recommendations, using multivariable logistic regression to control for other factors.

**Results:**

Three hundred and eleven women at a median of 32-weeks of gestation completed the survey, a response rate of 85.2%. Most women reported that if they were told they were at increased risk for PTB, they preferred not to use PTB prevention (65.8%), of whom almost all (93.4%) reported they preferred close-monitoring and 6.6% preferred neither monitoring nor prevention. A much smaller proportion of women reported that they would not follow their healthcare provider’s recommendation for progesterone (10.9%) compared to pessary (28.7%) or cerclage (50.2%). Women who were neither married nor in a common-law relationship were more likely to report that they would not follow recommendations for progesterone (aOR = 5.88 [95% CI: 1.72, 20.00]). Most women (84.5%) reported they would use other sources of information other than their main healthcare provider to learn more about PTB prevention, with the most popular source being the internet.

**Conclusions:**

Most women reported that if they were told they were at increased risk of PTB, they preferred close-monitoring over using PTB prevention. Their reported likelihood of not following their healthcare provider’s recommendations for PTB prevention varied from 10.9% for progesterone to 50.2% for cerclage. These findings suggest that more education about the risk of PTB, PTB preventions, as well as compliance with progesterone is needed and that the internet would be an important source of information. However as our study was completed by women at a median of 32 weeks of gestation, future surveys targeted at women earlier in their pregnancy are needed.

**Electronic supplementary material:**

The online version of this article (doi:10.1186/s12884-017-1221-z) contains supplementary material, which is available to authorized users.

## Background

Preterm birth (PTB) is a serious public health concern. It is the leading cause of perinatal mortality in Canada [1] and is also associated with an increased risk of neonatal death [2]. Beyond its contribution to mortality, PTB can have lifelong effects on neurodevelopmental functioning through cerebral palsy, impaired learning, and visual disorders [3]. The maternal psychological cost is also high as many women experience anxiety and stress over the immediate and long-term health consequences for their preterm infant [4].

Interventions to reduce the risk for PTB include progesterone [5], cerclage [6, 7], and pessary [8]. Most of these studies have been conducted in women with a short cervix and/or a previous spontaneous PTB [5–7]. Furthermore, to date, studies have not found one prevention to be most effective in reducing PTB risk [9, 10]. The uncertainty over the best prevention is reflected in guidelines which note the limited evidence to support progesterone [11] and cerclage uses [12]. In the absence of clear guidelines, the decision to use PTB prevention is mostly left up to women and their healthcare providers. Given that 56.2% of women who eventually have PTB do not have traditional PTB risk factors [13], studying the acceptability of PTB prevention and identifying preferences and concerns that can be addressed during counselling is important.

Moreover, although there is a call for patient-centred prenatal care [14], women’s preferences for and concerns about PTB prevention have not been well-studied. A previous cross-sectional survey [15] and two chart review studies [16, 17] focused on the acceptability of and concerns about progesterone. We identified one healthcare provider survey on cerclage [18], but most studies have focused on counselling practices on progesterone [15, 18–24]. The need to consider women’s preferences is important because evidence has shown that most women reported greater satisfaction with their pregnancy when they were actively involved in medical decisions [25], and satisfaction, in turn, has been linked to increased confidence in their ability to take better care of themselves and their infants [26]. Despite this importance, there has been no study to date conducted in women to understand their preferences for and concerns about PTB prevention overall.

To help address this important gap, we designed a survey, informed by previous studies [15] to understand women’s preferences for and concerns about PTB prevention including progesterone, cerclage, and pessary.

## Methods

The design of our survey followed Dillman’s Method [27] and the reporting of results was informed by Kelley et al. [28].

### Recruitment

The study sample included women from obstetric clinics and midwifery clinics in Southwestern Ontario, Canada. Between November 2015 and February 2016, clinic staff mentioned the survey to all potentially eligible women. Interested women were referred to research personnel (V.H.), who described the purpose of the survey and distributed the hard copies of the questionnaire. Consent to participate was indicated by completion and submission of the questionnaire. The Hamilton Health Sciences/McMaster University granted Research Ethics Board approval for study prior to commencement (Reference Number: 2015-0459-GRA).

### Eligibility Criteria

Pregnant women who could read English sufficiently well were eligible to participate. Because greater than 56.2% of women who experience PTB do not present with traditional risk factors [13], we included all women, not only those identified as having increased risk of PTB.

### Questionnaire

To inform the design of our questionnaire, the first to our knowledge, to study women’s preferences for and concerns about PTB prevention including progesterone, cerclage, and pessary, three electronic databases (MEDLINE, EMBASE, and CINAHL) were searched up until April 2015 to identify previous surveys on PTB prevention. One previous survey that used an invalidated questionnaire to ask women about their preferences for progesterone therapy was identified [15]. We used the findings of this previous study to inform the development of our questionnaire.

Our questionnaire gathered information on: 1) sociodemographics, 2) past and current pregnancies, 3) preferences for and concerns about each of the three PTB preventions, 4) likelihood of following their healthcare provider’s recommendation for each of the PTB preventions and 5) sources of information that women would seek to learn more about PTB prevention (see Additional File 1). Questions were a mix of five-point Likert scales, open-ended, and semi-closed ended questions. The questionnaire was piloted for comprehensibility of questions and answers and for flow in three women of child-bearing age and was revised accordingly.

### Outcomes

Our primary outcome was the preference *not* to use PTB prevention if women were told they were at increased risk for PTB. Secondary outcomes included the proportion of women who reported they would *not* follow their healthcare provider’s recommendation for PTB prevention and associated factors.

### Sample size calculation

To provide a precise estimate for our primary outcome, we used a one proportion calculation formula to determine our sample size: n = p (1-p) (1.96/m)^2^, where *n* is the sample size, *p* is the estimated proportion, and *m* is the margin of error. Because we were unsure of the proportion of women who would prefer any PTB prevention, we assumed that *p* = 0.50. For this estimated proportion, we found that the formula provided the largest sample size when *m* = 0.06. Thus, we conservatively estimated that we needed to recruit 310 respondents for our survey with a missing data rate of 15%.

### Statistical Analysis

Responses from completed questionnaires were entered into LimeSurvey (Version 1.91+ Build 120302) for data management. Data were analyzed using SAS 9.4 (SAS Institute Inc, Cary, North Carolina). Normally distributed continuous variables were calculated as means with standard deviations (SD) and non-normally distributed continuous variables were calculated as medians with their interquartile ranges (IQR). Categorical variables were described as counts and percentages. Continuous variables were tested for significance using the independent *t*-test or Mann–Whitney-Wilcoxon test and categorical variables were tested using Fisher’s Exact or chi-squared tests. A threshold of p ≤ 0.05 was used to determine statistical significance.

Multivariable logistic regression was conducted for our primary and secondary outcomes, and the results are reported as adjusted odds ratios (aOR) with 95% confidence intervals (CIs). To select variables for our multivariable logistic regression, we considered *a priori* biologically plausible variables performed with a p-value for entry in a stepwise regression set at 0.10 and p-value for stay set at 0.20. *A priori* chosen variables included age, ethnicity, education, relationship status, type of healthcare provider whom women selected for prenatal care, smoking status, PTB risk status for the current pregnancy, gestational week at the time the questionnaire was completed, concerns about PTB preventions, and in the case of the secondary outcome, preference to not use PTB prevention. Multicollinearity was assessed using variance inflation factor (VIF), where a VIF less than or equal to five was considered to be evidence of multicollinearity. If multicollinearity was detected, the variable with the most biological plausibility was kept or if biological plausibility between variables were considered equal, the variable with the greater number of responses was kept. Only participants who provided a response to every question in the questionnaire was included in the multivariable logistic model for analysis (i.e. complete-case analysis).

For our primary outcome, women were asked to “imagine a scenario in which their healthcare provider thought they were at increased risk of premature or early birth” and then to indicate which of the following types of management they would prefer. Based on their responses, we grouped women who responded with a preference for one or more of the three PTB preventions (progesterone, cerclage, pessary) as having a “preference to use prevention” while women who responded with a preference for close-monitoring only or preference for no prevention at all as having a “preference to *not* use prevention.” For our secondary outcome, women were asked to indicate “how likely or unlikely they would be to follow their healthcare provider’s recommendation if their main healthcare provider had recommended the following preventions?” Taking a similar approach as our primary outcome, women who reported that they were “not likely” or “slightly unlikely” to follow healthcare provider’s recommendation on a 5-point Likert scale were categorized as those who “reported they would not follow the recommendation” and women who reported that they were “neutral,” “slightly likely,” or “extremely likely” as those who “reported neutral or would follow recommendation.”

## Results

### Recruitment results

Of the 365 pregnant women who were eligible, 311 women completed the survey, a response rate of 85.2%. One woman was excluded as she had had >50% incomplete data. The average missing data rate per question was 1.4%.

### Characteristics of survey respondents

Respondents had a mean age of 30.9 years and self-identified predominantly as European/White-Caucasian (82.1%, Table 1). Most women were married or in a common-law relationship (94.5%), and had received at least some post-secondary education (84.5%). A small proportion currently smoked (7.4%). The median gestational age at the time the questionnaire was completed was 32.0 weeks (IQR: 26.7- 35.7 weeks). Most women had had a previous pregnancy (61.5%), of whom 25 (13.2%) reported that they had had a PTB. Thirteen (4.2%) pregnant women have been told by their healthcare provider that their current pregnancy was at increased risk for PTB, of whom 10 had received a cervical assessment by ultrasound only (76.9%), one received a cervical assessment by ultrasound and progesterone (7.7%), one was instructed to do light physical activity (7.7%), and one did not report receiving an ultrasound or advice (7.7%). Respondents reported receiving most of their prenatal care from obstetricians (44.4%), midwives (33.8%), and family doctors (17.4%).

**Table 1:** Characteristics of the study sample of survey respondents

Variable	Response, *n* (%)^a^
Pregnant Women’s Characteristics	
Age (years), mean ± SD	30.9 (5.4)
Gestational age (weeks), median (interquartile range)	32.0 (26.7, 35.7)
European/White-Caucasian (self-reported)	252 (82.1)
Married or in a common-law relationship	292 (94.5)
Education	
Secondary school or less	48 (15.5)
Post-secondary school	262 (84.5)
Current smokers	23 (7.4)
Obstetric History	
Prior miscarriage	28 (14.7)
Prior full-term birth (≥37 weeks)	150 (48.5)
Prior preterm birth (<37 weeks)	25 (13.2)
Received progesterone	2 (8.0)
Advised to rest in bed (bedrest)	1 (4.0)
Current Pregnancy Characteristics	
First-time pregnancy	119 (38.5)
Told by healthcare provider that they were at increased risk for preterm birth	13 (4.2)
Received ultrasound	10 (76.9)
Received ultrasound + progesterone	1 (7.7)

^a^ *N* = 311 respondents completed the survey

### Preference for PTB prevention

Most women (65.8%) reported that if their healthcare provider told them they were at increased risk for PTB, they would prefer not to use any prevention; of whom 185 (93.4%) women preferred close-monitoring only and 13 (6.6%) women preferred no prevention at all including close-monitoring (Table 2). Of those who did report a preference for prevention, 53 (60.2%) women reported they preferred to use one type of prevention to manage their PTB risk and 25 (24.3%) women reported they preferred to use all three preventions. Progesterone was the most frequently chosen choice amongst women who preferred to use one type of prevention (84.9%) and amongst women who preferred to use two preventions (94.4%). Our univariate and multivariate logistic regression did not identify any significant factors associated with a preference to *not* use PTB prevention.

**Table 2:** Comparison of the characteristics between women who preferred no prevention vs women who preferred at least one prevention

Variables^a^	Preferred no prevention^b^, (*n* = 198)	Preferred prevention^c^ (*n* = 103)	p-value for difference^d^
Pregnant Women’s Characteristics			
Age (years), mean ± SD	30.7 (5.6)	31.0 (5.1)	0.64
European or White-Caucasian ethnicity (self-reported)	167 (85.6)	90 (88.2)	0.59
Post-secondary education	164 (82.8)	90 (87.4)	0.32
Married or in common-law relationship	185 (93.9)	99 (96.1)	0.59
Current Smokers	14 (7.1)	8 (7.8)	0.82
Primary Healthcare Provider			
Obstetrician	87 (43.9)	49 (47.1)	0.42
Family physician	62 (31.3)	37 (35.6)	
Midwife	40 (20.2)	13 (12.5)	
Others^e^	9 (4.6)	5 (4.8)	
Obstetric History			
Prior full-term birth	95 (79.8)	51 (76.1)	0.58
Prior preterm birth	14 (11.8)	11 (16.2)	0.50
Prior use of preterm birth prevention	2 (14.3)	1 (9.1)	1.00
Received progesterone	0 (0.0)	1 (50.0)	1.00
Advised to rest in bed (bedrest)	1 (100.0)	1 (50.0)	1.00
Current Pregnancy Characteristics			
Gestational age at point of survey completion (weeks), mean ± SD^f^	31.0 (7.6)	29.6 (7.5)	0.12
At increased risk for preterm birth	12 (6.1)	1 (1.0)	0.04
Preterm Birth Prevention Preferences			
Preferred no close-monitoring nor prevention	13 (6.6)		
Preferred close-monitoring only	185 (93.4)		
Preferred close-monitoring and prevention		15 (14.6)	
Preferred progesterone only		13 (86.7)	
Preferred cerclage only		1 (6.7)	
Preferred all 3 preventions		1 (6.7)	
Preferred preterm birth prevention only		88 (85.4)	
Selected 1 prevention only		53 (60.2)	
Progesterone only		45 (84.9)	
Cerclage only		5 (9.4)	
Pessary only		3 (5.7)	
Selected 2 preventions		18 (20.4)	
Progesterone + Cerclage		8 (44.4)	
Progesterone + Pessary		9 (50.0)	
Cerclage + Pessary		1 (5.6)	
Selected all 3 preventions		25 (24.3)	

^a^Data is expressed as n (%) unless otherwise specified

^b^“Preferred no prevention” included women who preferred no prevention, close-monitoring only, or were not using a prevention if they were at increased risk for preterm birth

^c^“Preferred prevention” included women who preferred a prevention, close-monitoring and a prevention, or were using a prevention if they were at increased risk for preterm birth

^d^Significance was assessed using independent *t*-test for continuous variables and chi-squared test or Fisher’s exact test for categorical variables;

^e^Others included Maternal-Foetal Medicine Specialists, Nurses, and Fertility Specialists

^f^Mann-Whitney-Wilcoxon test was used to test for significance

Almost all women rated concerns about effectiveness of the three preventions or the potential harm caused by them to the infant or themselves as “slightly important” or “extremely important”, on a Likert scale ranging from “extremely unimportant” to “extremely important” (Table 3). In contrast, the cost of progesterone was not viewed as being of high importance to most women.

**Table 3:** Importance of various concerns about preterm birth prevention

Issue	Number of responses, *n*	Not at all important, *n* (%)	Slightly not important, *n* (%)	Neutral, *n* (%)	Slightly important, *n* (%)	Extremely important,*n* (%)
Potential Harm Caused to Baby						
Progesterone	309	1 (0.3)	1 (0.3)	7 (2.3)	8 (2.6)	292 (94.5)
Cerclage	306	2 (0.6)	2 (0.6)	4 (1.3)	9 (2.9)	289 (94.4)
Pessary	307	2 (0.6)	2 (0.6)	9 (2.9)	9 (2.9)	285 (92.8)
Potential Harm Caused to Mother						
Progesterone	309	4 (1.3)	6 (1.9)	20 (6.5)	86 (27.8)	193 (62.5)
Cerclage	306	5 (1.6)	5 (1.6)	14 (4.6)	60 (19.6)	222 (72.5)
Pessary	307	3 (1.0)	5 (1.6)	18 (5.9)	71 (23.1)	210 (68.4)
Effectiveness of Prevention						
Progesterone	309	1 (0.3)	4 (1.3)	29 (9.4)	67 (21.7)	239 (78.1)
Cerclage	306	2 (0.6)	3 (1.0)	22 (7.2)	40 (13.1)	239 (78.1)
Pessary	307	2 (0.6)	3 (1.0)	26 (8.5)	52 (16.9)	224 (73.0)
Associated Financial Cost						
Progesterone	309	92 (29.8)	38 (12.3)	75 (24.3)	65 (21.0)	39 (12.6)

### Likelihood of following healthcare provider’s recommendation for PTB prevention

The highest proportion of women reported they would not follow their healthcare provider’s recommendation wa﻿s for cerclage (50.2%), followed by pessary (28.7%) and then progesterone (10.9%). (Table 4)

**Table 4:** Comparison of charactertistics between women who reported unlikely vs women who reported undecided/likely to follow preterm birth prevention

Variables^a^	Progesterone			Cerclage			Pessary		
	Unlikely to follow recommendation^b^ (*n* = 27)	Undecided or likely to follow recommendation^c^ (*n* = 220)	p-value^d^	Unlikely to follow recommendation^b^ (*n* = 123)	Undecided or likely to follow recommendation^c^ (*n* = 122)	p-value^d^	Unlikely to follow recommendation^b^ (*n* = 70)	Undecided or likely to follow recommendation^c^ (*n* = 174)	p-value^d^
Pregnant Women’s Characteristics									
Age 30 and over	13 (48.1)	146 (66.4)	0.09	78 (63.4)	81 (66.4)	0.59	41 (59.4)	117 (67.2)	0.30
European or White- Caucasian ethnicity (self-reported)	23 (85.2)	197 (90.8)	0.32	109 (88.6)	108 (90.8)	0.67	58 (84.1)	159 (92.4)	0.06
Post-secondary education	23 (85.2)	190 (86.8)	0.77	104 (84.6)	106 (87.6)	0.58	57 (82.6)	153 (87.9)	0.30
Married or in common-law relationship	22 (81.5)	212 (96.8)	0.005	116 (94.3)	116 (95.9)	0.77	65 (94.2)	167 (96.0)	0.51
Current Smokers	3 (11.1)	18 (8.2)	0.71	9 (7.3)	13 (10.7)	0.38	4 (5.8)	17 (9.8)	0.45
Primary Healthcare Provider									
Obstetrician	12 (44.4)	80 (36.4)	0.32	42 (34.2)	50 (41.0)	0.26	27 (38.6)	64 (38.8)	0.52
Family physician	7 (25.9)	39 (17.7)		27 (22.0)	18 (14.8)		16 (22.9)	29 (16.7)	
Midwife	8 (29.6)	89 (40.4)		46 (37.4)	50 (41.0)		23 (32.9)	73 (42.0)	
Others^e^	0 (0.0)	12 (5.4)		8 (6.5)	4 (3.3)		4 (5.7)	8 (4.6)	
Obstetric History									
Prior full-term birth	13 (48.1)	107 (48.6)	0.13	58 (47.2)	60 (49.2)	0.84	32 (45.7)	86 (49.4)	0.46
Prior preterm birth	2 (7.4)	17 (7.7)	0.67	6 (4.9)	12 (9.8)	0.21	3 (7.9)	15 (13.9)	0.40
Prior use of preterm birth prevention	1 (50.0)	2 (11.8)	0.31	1 (16.7)	2 (16.7)	1.00	0 (0.0)	3 (20.0)	1.00
Current Pregnancy Characteristics									
Third trimester at point of survey completion	17 (63.0)	155 (70.8)	0.50	87 (71.3)	84 (68.8)	0.78	47 (67.1)	123 (71.1)	0.54
Increased risk for preterm birth	1 (3.7)	9 (4.1)	1.00	2 (1.6)	7 (5.7)	0.10	0 (0.0)	9 (5.2)	0.06
Preterm Birth Prevention Preferences and Issues									
Preferred no prevention	20 (76.9)	137 (63.7)	0.20	96 (78.7)	61 (52.1)	<0.0001	56 (80.0)	100 (59.5)	0.003
Likely to have concerns about potential harm to baby	27 (100.0)	213 (96.8)	1.00	118 (95.9)	118 (96.7)	1.00	66 (94.3)	165 (94.8)	1.00
Likely to have concerns about potential harm to mother	23 (85.2)	201 (91.4)	0.29	111 (90.2)	112 (91.2)	0.82	62 (88.6)	158 (90.8)	0.64
Likely to have concerns about efficacy	25 (92.6)	196 (89.1)	0.75	112 (91.1)	111 (91.0)	1.00	60 (85.7)	158 (90.8)	0.26
Likely to have concerns about cost	13 (48.2)	67 (30.4)	0.08						

^a^Data expressed as n (%) unless otherwise specified; ^b^“Unlikely to follow recommendation” included women who selected not very likely or slightly unlikely to follow healthcare provider’s recommendation; ^c^“Undecided or likely to follow recommendation” included women who selected neutral, slightly likely, or very likely to follow healthcare provider’s recommendation; ^d^Significance was assessed with independent *t*-test for continuous variables and chi-squared test or Fisher’s exact test for categorical variables; ^e^Others included Maternal-Foetal Medicine Specialists, Nurses, and Fertility Specialists

Women who were not married or in a common-law relationship were significantly more likely to report they would *not* follow recommendations for progesterone (aOR = 5.88 [95% CIs: 1.72, 20.00]; p = 0.0049, Table 5) than women who are married or in a common-law relationship. In addition, women who preferred not to use prevention were more likely to report they would not follow recommendations for cerclage (aOR = 3.39 [95% CI: 1.93, 5.96]; p < 0.0001) or pessary (aOR = 2.99 [95% CI: 1.51, 5.92]; p = 0.002).

**Table 5:** Multivariate logistic regression of the likelihood of not following healthcare provider’s recommendation

Variables^a^	Progesterone		Cerclage		Pessary	
	Multivariate Analysis OR (95% CIs)	p-value^b^	Multivariate Analysis OR (95% CIs)	p-value^b^	Multivariate Analysis OR (95% CIs)	p-value^b^
Pregnant Women’s Characteristics						
European or White-Caucasian ethnicity (self-reported)					0.41 (0.17, 1.01)	0.05
Not married or in common-law relationship	5.88 (1.72, 20.00)	0.0049				
Preterm Birth Prevention Issues^c^						
Preference for no prevention			3.39 (1.93, 5.96)	<0.0001	2.99 (1.51, 5.92)	0.002

Abbreviations: *OR*, odds ratio; *CIs*, confidence intervals. ^a^“Preferred no prevention” included women who preferred no prevention or close-monitoring or were not using a prevention if they were at increased risk for preterm birth; ^b^Significance was assessed using chi-squared test

### Sources of information on PTB prevention

Two hundred sixty-two women (84.5%) reported they would use other sources of information other than their main healthcare provider to help them make their decisions to use PTB prevention. The top four information sources that women reported they would use were the internet (70.9%), family and friends (55.5%), other healthcare providers (40.0%), and books or scientific literature (38.1%).

## Discussion

This was the first survey to our knowledge conducted to study the preferences for and concerns about current options for preterm prevention in pregnant women. Most women reported that they preferred not to use prevention but preferred close-monitoring of their pregnancy if they were told by their healthcare provider they were at increased risk for PTB. Approximately 50.2% and 28.7% of women reported that they would not follow their healthcare provider’s recommendations for cerclage and pessary, respectively, but only 10.9% did for progesterone. These findings were surprising because we had hypothesized that pessary would be more attractive than progesterone to many women and since previous surveys have reported a high acceptance rate for PTB prevention with progesterone among women who had been identified to be at increased risk of PTB [15–17].

There are a few reasons which might explain why most women preferred not to use prevention if they had been told by their healthcare provider they were at increased risk of PTB. Although it may relate to the hypothetical nature of the scenario, it may also reflect respondents’ lack of understanding about the risks of preterm birth, which points to important opportunities for education. Alternatively, healthcare providers’ satisfaction with their knowledge and confidence in the quality of evidence for progesterone have been associated with its prescription [24]. Interestingly, patient characteristics do not consistently elicit its prescription, even for women visiting the same clinic [14, 24]. Other studies also reported that there is significant variation in clinical practice in progesterone for PTB prevention even amongst those working in the same clinic serving women with high-risk pregnancies [14, 16–18]. However, two surveys found that most healthcare providers would be willing to take part in hypothetical progesterone trials to further evaluate its effects on PTB prevention [21, 29]. Taken together these studies suggest that the behaviour of healthcare providers may play an important role in women’s decision to use PTB prevention and healthcare provider’s preferences for and concerns about PTB prevention need to be studied to better understand how their thought processes may influence their clinical practice.

The reluctance to follow recommendations for preventions that are more intrusive may be due to concerns about complications. In addition, we found that women who were not married or in a common-law relationship were more likely to report that they would not follow recommendations for progesterone. Marriage may be a proxy for social support [30] and less immediate social support may impact decision making. Previous studies have reported that women who are not married were more likely to start prenatal care later and have fewer prenatal visits [31] and in turn, women who receive less prenatal care have been associated with lower self-esteem and confidence [32]. Providing additional support or resources might be particularly important to this group of women, hence suggesting reputable online information sources would likely be beneficial.

Strengths of our study include a diverse sample of women from a broad range of healthcare providers including obstetricians, midwives and family physicians. Second, the response rate of our survey of 85.2% is robust, thus reducing the potential impact of selection bias. Third, our questionnaire was piloted, which likely increased read-ability. Finally, we used previous surveys that studied the preferences and values for PTB prevention to inform the design of our questionnaire [15].

Limitations of our study include that most women were relatively well educated, therefore, our results can only be understood in this context. Women who are more educated may prefer active participation in medical decisions [33] and are less likely to rely solely on the expertise of their healthcare providers for information. Further, because women in our sample were at a median of 32 weeks of gestation, they may prefer not to use PTB prevention because they were in the third trimester, and unaware of the risks of giving birth at this point in the pregnancy. Future surveys should consider women earlier in their pregnancy. Second, most women who responded to our survey were not at increased-risk for PTB. Hypothetical scenarios represent abstractions of real-life phenomena and women who are not at increased risk for PTB may answer differently if they were actually at increased risk. However, it is also possible that because our questionnaire offers anonymity and confidentiality, women may be more likely to answer their actual preferences for PTB prevention. Previous studies have found that almost one-fifth of women using progesterone to manage risk of PTB report issues of compliance [17]. More research needs to be conducted around patient compliance with treatment.

## Conclusions

Our findings have implications for women’s prenatal care. We found that most women preferred not to use PTB prevention but still preferred close-monitoring of their pregnancy if they were told by their healthcare provider they were at increased risk for PTB. A sizeable portion of women reported that they would not follow their healthcare provider’s recommendations for cerclage and pessary, respectively, hence, providing additional education to women requiring such interventions would be particularly important. Since the complications of PTB can be severe and long-lasting, it is important to provide high quality education to women about PTB and the benefits associated with PTB prevention, including through websites as 70% of women in our survey reported they would use the internet to learn more about PTB prevention. Written or online information could help supplement the education provided by healthcare providers, which is important as a previous study had reported poor recall concordance between physicians and parents on key factors like potential neonatal complications resulting from PTB [34]. Further, because of the importance of healthcare providers influence on prenatal care, there is a need to better understand the healthcare providers’ knowledge of, preferences for, and concerns about PTB prevention.

## Additional file

Additional file 1: Questionnaire adminstered to women to assess their thoughts about and preferences for PTB prevention. (PDF 578 kb)

## Abbreviations

aOR: Adjusted odds ratios
CIs: Confidence intervals
IQR: Interquartile ranges
m: Margin of error
n: Sample size
p: Estimated proportion
PTB: Preterm birth
SD: Standard deviation
VIF: Variance inflation factor
