# Supplementary material for: Pregnant women’s preferences for and concerns about preterm birth prevention: a cross-sectional survey
Source: BMC Pregnancy Childbirth. 2017 Jan 31;17:49. doi: 10.1186/s12884-017-1221-z (PMC5286823; doi:10.1186/s12884-017-1221-z)
Supplement: Additional file 1: — Questionnaire adminstered to women to assess their thoughts about and preferences for PTB prevention. (PDF 578 kb) [file 12884_2017_1221_MOESM1_ESM.pdf]

A STUDY ON PREMATURE OR EARLY BIRTH PREVENTION:

A SURVEY FOR PREGNANT WOMEN

We invite you to share your thoughts on interventions used for premature or early birth prevention.

Your participation is very important and greatly appreciated. This survey will help us understand the thoughts and preferences on interventions used for premature or early birth prevention. The information gathered will hopefully be able to help improve healthcare received by women and the health of women and children.

This study is being conducted by researchers at McMaster University.
The survey will take only about 10-15 minutes to complete.

Your participation is completely voluntary, and you are free to withdraw at any time by not submitting your survey. If you decide to participate, please complete the survey and return it to the drop-off box. By returning the survey you are giving your consent to participate in the study. Please understand that your answers will be **completely confidential,** and there will be no way of identifying participants. **Please do not write any identifying information on this survey (e.g., name, address, or phone number).**

Should you have any questions about this study, please contact Dr. Sarah McDonald at (905) 525-9140 ext. 26622. **This study has been reviewed and approved by the Research Ethics Board at McMaster University.** If you have any questions or concerns about your rights as a research participant, please feel free to contact the Hamilton Health Sciences/Faculty of Health Sciences Research Ethics Board at (905) 521-2100 ext. 42013.

REB Approval Number: 2015- 0459- GRA

Thank you for your participation.

Today's date: _______ _______ ____________
month **day** **year**

SECTION I- BASIC INFORMATION

We would like to understand more about the people who are answering our questionnaire. Please let us learn more about you.

1. **What is your age?**

   ______ years

2. **What is your ethnicity?** *(Please check ALL THAT APPLY)*.

   - ☐ African
   - ☐ Asian
   - ☐ European/Caucasian
   - ☐ Hispanic
   - ☐ Middle Eastern
   - ☐ Native American
   - ☐ Unknown
   - ☐ Other (*Please specify):* ___________________

3. **What is the highest level of education you received?**

   - ☐ Less than high school
   - ☐ High school (completed)
   - ☐ College
   - ☐ University

4. **What is your current relationship status?**

   - ☐ Married/Common law
   - ☐ Single/ Widowed/ Separated/Divorced

5. **Do you currently smoke?**

   - ☐ No
   - ☐ Yes → **Are you on smoking cessation therapy (eg. nicotine patch)?**
     - ☐ No
     - ☐ Yes

SECTION II- PREVIOUS PREGNANCY

Please tell us about your previous pregnancy. Premature or 'early' birth is when a baby is born before 37 weeks (generally, a 'due date' is considered about 40 weeks).

6. **Have you ever been pregnant before?**

   - ☐ No → **Please skip to question 8 (section III).**
   - ☐ Yes → **How many pregnancies went to full term (at least 37 weeks)?** (*Please fill in the blank. Note that twins count as one pregnancy).*

     ______ pregnancy (or pregnancies)

7. **Have you had a premature or early birth before 37 weeks?**

- ☐ No → **Please skip to question 8 (section III).**
- ☐ Yes → **How many pregnancies were premature or early births?** (*Please fill in the blank. Note that twins count as one pregnancy)*
  ______ pregnancy (or pregnancies)

  → **Did you receive any intervention (eg. progesterone or cerclage) from your healthcare provider to decrease the chances of premature or early birth?**
  - ☐ No
  - ☐ Yes → (*Please specify the intervention in the blank space below).*
    ____

SECTION III- CURRENT PREGNANCY

Please tell us about your current pregnancy.

8. **For this pregnancy, how many baby (or babies) are you expecting?**
   _______ baby (or babies)

9. **When is your due date?**

   _______ _______ ______________
   month day year

10. **During this pregnancy, who has provided most of your healthcare so far? (***Please check ONE ONLY).*
    - ☐ Maternal-Fetal Medicine Specialist
    - ☐ Obstetrician
    - ☐ Midwife
    - ☐ Family Doctor
    - ☐ Nurse
    - ☐ Other (*Please specify)*: __________________

11. **Has your healthcare provider told you that your current pregnancy is at increased risk for premature or early birth (before 37 weeks)?**

- ☐ No → **Please go to question 12.**
- ☐ Yes → **Are you currently using any of the following?** (*Please check ALL THAT APPLY*).
  - ☐ Ultrasounds (to measure the length of the cervix, opening of the uterus)
  - ☐ Progesterone (a medication given to try to prevent premature or early birth)
  - ☐ Cerclage (surgery to sew the cervix or the opening of the uterus closed to try to prevent premature or early birth)
  - ☐ Pessary (a plastic ring that keeps cervix or the opening of the uterus closed to try to prevent premature or early birth)
  - ☐ Other (*please specify*): ___________________

→ **Once you have completed question 11, <u>please skip to question 13.</u>**

12. **Please only answer this question if you are NOT at risk for premature or early birth. Please imagine a scenario in which your healthcare provider thought you were at increased risk of premature or early birth. Which of the following care would you prefer?**

- ☐ Not prefer any intervention (including no increase in the closeness of monitoring)
- ☐ Prefer close monitoring only
- ☐ Prefer an intervention
  - → **Which intervention would you prefer?** (*Please check ALL THAT APPLY*).
    - ☐ Progesterone (a medication given to try to prevent premature or early birth)
    - ☐ Cerclage (surgery to sew the cervix or the opening of the uterus closed to try to prevent premature or early birth)
    - ☐ Pessary (a plastic ring that keeps the cervix or the opening of the uterus closed to try to prevent premature or early birth)
    - ☐ Other (*Please specify*): ___________________

13. **If your main healthcare provider had recommended one of the following interventions, how likely or unlikely would you be to follow the recommendation?** (*Please place an "X" in the appropriate column for each prevention*).

	Extremely likely	Slightly likely	Neutral	Slightly unlikely	Extremely unlikely
Progesterone					
Cerclage					
Pessary					

SECTION IV- ATTEMPTS TO PREVENT PREMATURE OR EARLY BIRTH

Please imagine you are at increased risk for premature or early birth before 37 weeks (even if you are not).

14. **When considering progesterone, a medication given to try to prevent premature or early birth, how important or unimportant are each of the following issues in influencing your decision?** (*Please place an "X" in the appropriate column for each issue*).

	Extremely important	Slightly important	Neutral	Slightly not important	Not at all important
Concerns about potential harm to the baby					
Concerns about potential harm to you					
Whether the medication works very well					
Whether there would be a cost for you					

15. **When considering the cerclage, surgery to sew the cervix or the opening of the uterus closed to try to prevent premature or early birth, how important or unimportant are each of the following issues in influencing your decision?** (*Please place an "X" in the appropriate column for each issue*).

	Extremely important	Slightly important	Neutral	Slightly not important	Not at all important
Concerns about potential harm to the baby					
Concerns about potential harm to you					
Whether the cerclage works well					

16. **When considering the pessary, a plastic ring that keeps cervix or the opening of the uterus closed to try to prevent premature or early birth, how important or unimportant are each of the following issues in influencing your decision?** (*Please place an "X" in the appropriate column for each issue*).

	Extremely important	Slightly important	Neutral	Slightly not important	Not at all important
Concerns about potential harm to the baby					
Concerns about potential harm to you					
Whether the pessary works well					

17. Other than your main healthcare provider, are there other sources of information you would use to help you make decisions regarding whether to use premature or early birth prevention?

- ☐ No
- ☐ Yes → **Which resource(s) would you use?** (*Please check ALL THAT APPLY).*
  - ☐ Books/Magazine
  - ☐ Family/Friends
  - ☐ websites/online communities/forums
  - ☐ Other healthcare providers (eg. pharmacist)
  - ☐ Television
  - ☐ Health Classes/Lectures
  - ☐ Other (*Please specify)*: ______________________

Thank you for completing this survey!

If there are any comments about this survey or on preterm birth prevention you would like to make, please share them with us in the box below.
